# Guidance for umbrella reviews of observational studies: A scoping review

**DOI:** 10.1002/jcv2.70017

**Published:** 2025-08-01

**Authors:** Carl Zhou, Nicholas Fabiano, Arnav Gupta, Stanley Wong, Kelly D. Cobey, David Moher, Sanam Ebrahimzadeh, Jeremy Y. Ng, Elena Dragioti, Jae Il Shin, Joaquim Radua, Samuele Cortese, Beverley Shea, Nicola Veronese, Lisa Hartling, Michelle Pollock, Stefania Papatheodorou, John P. A. Ioannidis, Marco Solmi

**Affiliations:** ^1^ Department of Psychiatry University of Ottawa Ottawa Ontario Canada; ^2^ Department of Medicine University of Calgary Calgary Alberta Canada; ^3^ College of Public Health Kent State University Kent Ohio USA; ^4^ Department of Psychiatry University of Toronto Toronto Ontario Canada; ^5^ Faculty of Medicine School of Epidemiology and Public Health University of Ottawa Ottawa Ontario Canada; ^6^ University of Ottawa Heart Institute Ottawa Ontario Canada; ^7^ Ottawa Hospital Research Institute Ottawa Ontario Canada; ^8^ Centre for Journalology Ottawa Hospital Research Institute Ottawa Ontario Canada; ^9^ Department of Nursing Research Laboratory Psychology of Patients, Families, and Health Professionals School of Health Sciences University of Ioannina Ioannina Greece; ^10^ Department of Pediatrics Yonsei University College of Medicine Seoul South Korea; ^11^ Severance Underwood Meta‐Research Center Institute of Convergence Science Yonsei University Seoul South Korea; ^12^ Imaging of Mood and Anxiety Related Disorders (IMARD) Group d'Investigacions Biomèdiques August Pi i Sunyer (IDIBAPS) CIBERSAM ES Barcelona Spain; ^13^ Department of Psychosis Studies Institute of Psychiatry Psychology and Neuroscience King's College London London UK; ^14^ Department of Medicine University of Barcelona Barcelona Spain; ^15^ Faculty of Environmental and Life Sciences Developmental EPI (Evidence Synthesis, Prediction, Implementation) Lab Centre for Innovation in Mental Health School of Psychology University of Southampton Southampton UK; ^16^ Faculty of Medicine Clinical and Experimental Sciences (CNS and Psychiatry) University of Southampton Southampton UK; ^17^ Hampshire and Isle of Wight Healthcare NHS Foundation Trust Southampton UK; ^18^ Hassenfeld Children's Hospital at NYU Langone New York University Child Study Center New York New York USA; ^19^ DiMePRe‐J‐Department of Precision and Rigenerative Medicine‐Jonic Area University of Bari “Aldo Moro” Bari Italy; ^20^ Geriatric Unit Department of Internal Medicine and Geriatrics University of Palermo Palermo Italy; ^21^ Department Pediatrics Faculty of Medicine & Dentistry Alberta Research Centre for Health Evidence University of Alberta Edmonton Alberta Canada; ^22^ Institute of Health Economics Edmonton Alberta Canada; ^23^ Department of Biostatistics and Epidemiology Rutgers School of Public Health Piscataway New Jersey USA; ^24^ Department of Epidemiology Harvard TH Chan School of Public Health Boston Massachusetts USA; ^25^ Department of Medicine Stanford University Stanford California USA; ^26^ Department of Epidemiology and Population Health Stanford University Stanford California USA; ^27^ Meta‐Research Innovation Center at Stanford (METRICS) Stanford University Stanford California USA; ^28^ Department of Mental Health The Ottawa Hospital Ottawa Ontario Canada; ^29^ Department of Child and Adolescent Psychiatry Charité Universitätsmedizin Berlin Germany

**Keywords:** evidence‐based medicine, evidence synthesis, meta review, observational study, overview of reviews, reporting guideline, systematic review, umbrella review

## Abstract

**Background:**

Umbrella reviews, or overviews of reviews, synthesize information using systematic reviews (SRs) as their unit of analysis. Although a formal guideline exists for reporting umbrella reviews of healthcare interventions (i.e. Preferred Reporting Items for Overviews of Reviews [PRIOR]), no formal guideline exists for conducting and/or reporting umbrella reviews of observational studies that examine epidemiological associations.

**Objective:**

To review the existing guidance on conducting and/or reporting umbrella reviews of observational studies on epidemiological associations, as part of the process of developing a formal reporting guideline.

**Methods:**

We reviewed the scoping review conducted in the context of PRIOR development and identified documents through forward citation search in PubMed, Scopus, and manual search in Google Scholar, Google Search up to December 22, 2024. Documents, regardless of format, were included if they provided guidance for conducting and/or reporting umbrella reviews of observational studies (including meta‐research studies of their features). Title/abstract screening and data extraction were performed independently and in duplicate and summarized narratively by stages of the umbrella review process.

**Results:**

The search retrieved 4491 unique records, with 96 full texts assessed and eight documents included. These documents, published between 2014 and 2023, offered guidance across seven topic areas, but overall guidance on conducting and/or reporting is limited. These areas include the answerable questions, prerequisite considerations, the scope of umbrella reviews, searching for SRs, primary data collection, analysis, presentation, and assessing the certainty/quality of the body of evidence.

**Conclusion:**

There is a need for dedicated, practical, and evidence‐based formal reporting guidelines for umbrella reviews of observational studies on epidemiological associations. This review lays the groundwork for developing the PRIOR‐extension for such studies: the Preferred Reporting Items for Umbrella Reviews of Cross‐sectional, Case‐control, and Cohort Studies.

## Graphical abstract

This review examined existing guidance on conducting and reporting umbrella reviews of observational studies on epidemiological associations, aiming to help develop a formal reporting guideline. The search identified 4491 records, with eight documents providing limited guidance across seven topic areas. The findings highlight the need for dedicated guidelines, leading to the development of the Preferred Reporting Items for Umbrella Reviews of Cross‐sectional, Case‐control, and Cohort Studies.

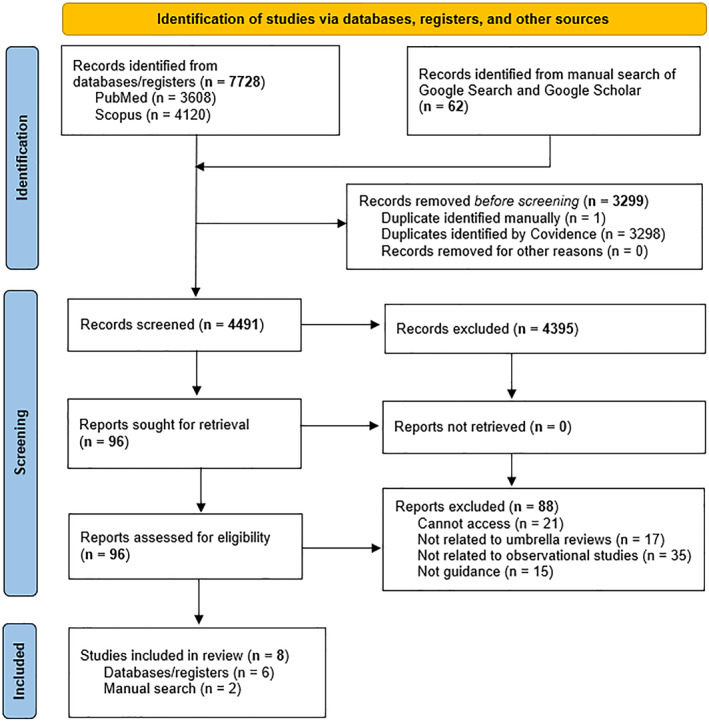

**Key points**

- Umbrella reviews synthesize findings from systematic reviews and are increasingly used to examine epidemiological associations using observational studies.
- Currently, there is no formal guideline for conducting or reporting umbrella reviews that focus on cross‐sectional, case‐control, and cohort studies.
- This scoping review identifies and summarizes existing but fragmented guidance across eight publications.
- Key gaps remain in areas such as protocol development, managing overlapping reviews, and assessing certainty of evidence.
- Relevance: This work supports the development of the Preferred Reporting Items for Umbrella Reviews of Cross‐sectional, Case‐control, and Cohort Studies reporting guideline, which aims to enhance methodological transparency and rigor in future umbrella reviews, thereby improving the quality of scientific evidence that informs policy and practice.

## INTRODUCTION

The proliferation of systematic reviews (SRs) and meta‐analyses (MAs) in the literature has escalated dramatically over the past 2 decades (Arango et al., 2021; Janiaud et al., 2021). This surge has given rise to a novel category of knowledge synthesis whereby a review is conducted on multiple SRs or MAs to consolidate their findings, which is known as “umbrella review,” “overview of (systematic) reviews,” “meta‐review,” or “review of reviews.” (Ioannidis, 2009; Papa and theodorou, 2019; Pollock et al., 2019) These reviews, which can encompass SRs of experimental, observational, or other study designs, aim to provide a comprehensive synthesis of information by using SRs or MAs as their unit of analysis (Pollock et al., 2019). This approach has become increasingly popular, as evidenced by the approximately eighteen‐fold increase in published umbrella reviews per year from 56 in 2010 to 1013 in 2024 (PubMed, [umbrella review]). In comparison, there was an approximately twelve‐fold increase in published SRs and meta‐analyses per year from 2291 in 2010 to 28,186 in 2024 (PubMed, [SR and meta‐analysis]), and a three‐fold increase in published cross‐sectional studies, case‐control studies, and cohort studies per year from 64,462 in 2010 to 190,734 in 2024 (PubMed, [cross‐sectional OR case‐control OR cohort]).

Overviews of (systematic) reviews of observational studies on epidemiological associations specifically have largely been reported as “umbrella reviews” in the literature (Arango et al., 2021; Janiaud et al., 2021). For consistency, we use the term “umbrella review” herein when referring to this study design.

Despite their growing popularity, the methodologies employed in umbrella reviews exhibit considerable variability, complicating the comparability of results (Arango et al., 2021; Solmi et al., 2018a). Although the Preferred Reporting Items for Systematic Reviews and Meta‐Analyses (PRISMA) guideline exists as the standard for reporting SRs and MAs (Page et al., 2021), heterogeneity persists, reflecting the diverse methodologies used in individual SRs and MAs (Hailes et al., 2019; Ioannidis, 2016; Solmi et al., 2018b). Further, the quality of evidence synthesis efforts is often fundamentally linked to the quality of the included studies within SRs and MAs. Generally, randomized controlled trials (RCTs) are regarded as higher‐quality sources within the evidence‐based medicine hierarchy. In contrast, observational studies, such as cross‐sectional, case‐control, and cohort studies, are often more susceptible to various biases (Ioannidis and Trikalinos, 2007).

To address these challenges, reporting guidelines have been developed to enhance the transparency and quality of research reporting (Moher et al., 2010). Notable examples include the PRISMA guidelines for SRs and MAs of interventions and the STROBE (Strengthening the Reporting of Observational Studies in Epidemiology) guidelines for observational studies (Page et al., 2021; von Elm et al., 2007). These guidelines, tailored to different study designs, provide structured checklists of essential information to report in a manuscript, thereby helping researchers standardize their reporting. This standardization facilitates the assessment of quality, reliability, and validity of research findings and enhances the reproducibility of studies. However, while reporting guidelines exist for umbrella reviews focused on SRs of healthcare interventions, that is, the Preferred Reporting Items for Overviews of Reviews (PRIOR) (Gates et al., 2022), guidance is lacking for those focusing on SRs of observational studies on epidemiological associations. Currently, no EQUATOR Network guidelines address the specific challenges of umbrella reviews for cross‐sectional, case‐control, and cohort studies. This gap highlights the need for tailored reporting guidelines to ensure the quality and reliability of such reviews.

In response to this gap, we are developing a reporting guideline for umbrella reviews for three types of observational studies: cross‐sectional, case‐control, and cohort. Following the EQUATOR guidance for developing reporting guidelines (Moher et al., 2010), we must identify existing knowledge to inform the development of our guideline, the Preferred Reporting Items for Umbrella Reviews of Cross‐sectional, Case‐control, and Cohort Studies (PRIUR‐CCC). Therefore, our goal with this study is to conduct a scoping review of existing documents that discuss the conduct and/or reporting of umbrella reviews of observational studies on epidemiological associations.

## METHODS

This scoping review was conducted in accordance with the Joanna Briggs Institute (JBI) methodology for scoping reviews (Peters et al., 2024). We followed the Preferred Reporting Items for SRs and Meta‐Analyses Extension Statement for Reporting of Scoping Reviews (PRISMA‐ScR) to guide our conduct, recognizing that PRISMA‐ScR is a reporting guideline (Tricco et al., 2018). This review is Step 2 of the EQUATOR guidance‐compliant project titled “Preferred Reporting Items for Umbrella Reviews of Cross‐sectional, Case‐control, and Cohort Studies (PRIUR‐CCC),” which aims to develop a reporting guideline for umbrella reviews of observational studies on epidemiological associations. The protocol of this project has been published (Solmi et al., 2024) and is publicly available at medrxiv.org (MEDRXIV/2022/283,572). The Ottawa Health Science Network Research Ethics Board (20220639‐01H) has approved the broader PRIUR‐CCC project, which includes the following components: (1) stakeholder identification, (2) a scoping review on umbrella review guidance, (3) a three‐round Delphi study, and (4) PRIUR‐CCC statement finalization.

### Search strategy

We reviewed the scoping review conducted in the context of PRIOR's development (Gates et al., 2020) for available guidance regarding the conduct and/or reporting of umbrella reviews of observational studies. The PRIOR scoping review included documents that met the broad definition of “being about overviews” or “discussing some aspect of overviews” in its title/abstract screening phase. Then, we performed forward citation searching in PubMed and Scopus for each document included in the PRIOR scoping review to identify “citing” references in Scopus or “similar articles” in Pubmed from 2020 (inclusive) (year of last search for the PRIOR scoping review (Gates et al., 2020)) through December 22, 2024. Manual searches were conducted using Google Scholar for academic literature and Google Search for gray literature during this time to supplement the comprehensive manual search conducted during the PRIOR scoping review (i.e., the websites of 59 organizations and major evidence synthesis centers up to February 12, 2019, the proceedings from four international conferences up to February 5, 2020). Manual searches used the search terms “umbrella review,” “overview of reviews,” “overview of SRs,” and “meta‐review” in conjunction with “guidance.” The first 20 results pages were reviewed, limited to articles since 2020.

### Inclusion/exclusion criteria

We included documents in any language or year that provided guidance applicable to the context or process of any aspect of conducting and/or reporting umbrella reviews of three types of observational studies (cross‐sectional, case‐control, and cohort studies), with or without empirical meta‐research studies addressing features of umbrella reviews. This can include documents not explicitly related to umbrella reviews of observational studies, such as those primarily focusing on experimental studies, diagnostic test accuracy, and prediction models.

We excluded guidance documents whose guidance was not applicable to the context or process of any aspect of conducting and/or reporting umbrella reviews of the aforementioned three types of observational studies. We excluded documents whose contents were unrelated to discussing methods or reporting guidance. We also excluded protocols, preprints, and conference abstracts for which a full version of the document that provided additional information was not available.

### Study screening and data extraction

Documents from the forward citation database search and all documents and abstracts cited in the PRIOR scoping review on guidance for overviews of reviews of healthcare interventions (Gates et al., 2020) were imported into Covidence, a SR management platform (Covidence Systematic Review Software, Veritas Health Innovation). Three reviewers (NF, AG, SW) independently screened titles and full texts and in duplicate, with disagreements resolved by consensus or the involvement of a third reviewer.

Two reviewers (among NF, AG, SW) independently extracted relevant data from the included literature onto a Microsoft Excel extraction form designed a priori. The data extraction form was not revised during the data extraction process. All extractions were done independently and in duplicate, with discrepancies resolved by consensus. The extracted guidance was narratively summarized and organized by stages of the umbrella review process. Specifically, the guidance was divided into the 15 topic areas identified by PRIOR (Gates et al., 2020), if applicable.

## RESULTS

**FIGURE 1:**
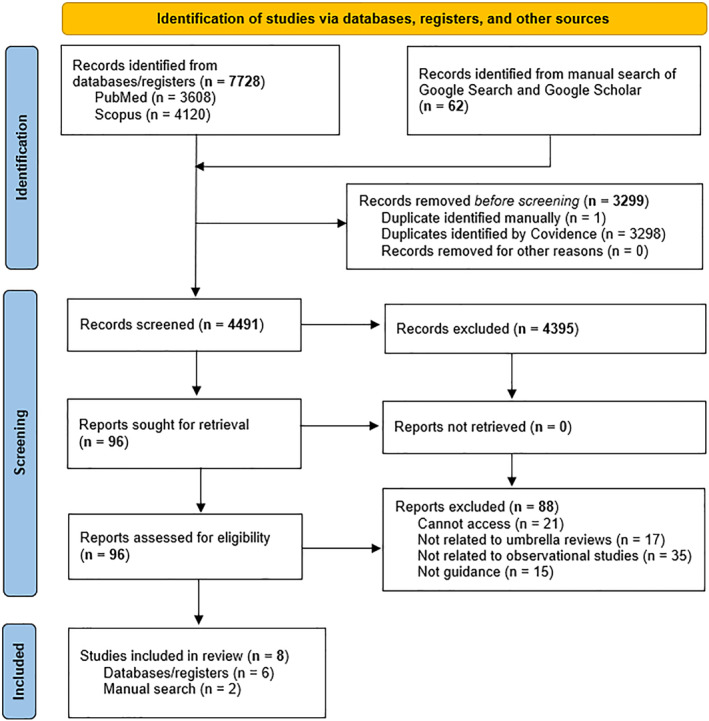
Preferred reporting items for systematic reviews and meta‐analyses flow diagram of document selection.

The initial searches retrieved 7790 records. 4491 unique records were retrieved after removing 3299 duplicates. We screened these records by title and abstract, excluding 4395. We then assessed the full text of 96 potentially relevant articles, excluding 88, and included eight documents published between 2014 and 2023. Figure 1 contains a flow diagram of the review process.

### Descriptive summary

The eight included documents comprise seven review articles (Arango et al., 2021; Aromataris et al., 2015; Baker et al., 2014; Belbasis et al., 2022; Bougioukas et al., 2023; Fusar‐Poli and Radua, 2018; Janiaud et al., 2021) and one letter to the editor (Gianfredi et al., 2022). Most review articles were narrative reviews, except for two reviews of umbrella reviews (Arango et al., 2021; Janiaud et al., 2021) and one meta‐research empirical study (Bougioukas et al., 2023). All documents provided guidance applicable to umbrella reviews of observational studies. Table 1 summarizes the characteristics of the eight included guidance documents. In the following sections, we summarize the available guidance for conducting and/or reporting umbrella reviews of observational studies subdivided into seven of the 15 topic areas identified in the PRIOR scoping review on guidance for overviews of healthcare interventions (Gates et al., 2020). We did not find guidance regarding the remaining eight topic areas, namely: (1) choosing between conducting an umbrella review of observational studies and a new SR, (2) author team composition and roles, (3) target audience of the umbrella review of observational studies, (4) developing and registering an umbrella review of observational studies' protocol, (5) collecting and presenting data on descriptive characteristics of included SRs (and their primary studies), (6) consideration and management of overlapping SRs, (7) interpreting outcome data and drawing conclusions, and (8) updating the umbrella review. The summarized guidance is presented in tabular format in Table 2.

**TABLE 1:** Characteristics of the included guidance documents (*n* = 8).

Author team (research group)	Year of publication	Document format
Bougioukas et al. (2023)	2023	Review article
Aromataris et al. (2015); (Joanna Briggs Institute)	2015	Review article
Baker et al. (2014)	2014	Review article
Belbasis et al. (2022)	2022	Review article
Fusar‐Poli and Radua (2018); (Department of Psychosis Studies, Institute of Psychiatry, Psychology and Neuroscience, London, UK)	2018	Review article
Gianfredi et al. (2022); (School of Medicine, Vita‐Salute San Raffaele University, Milan, Italy)	2022	Letter
Arango et al. (2021)	2021	Review article
Janiaud et al. (2021); (Meta‐Research Innovation Center at Stanford [METRICS], Stanford, USA)	2021	Review article

**TABLE 2:** Summary of the available guidance‐related information for conducting and/or reporting umbrella reviews of observational studies.

Topic	Reporting authors	Summary of reported guidance
What types of questions can be answered using the umbrella review format?	Bougioukas et al. (2023)	Umbrella reviews of observational studies typically examine the link between exposures and disease development, investigating risk factors and biomarkers using various observational study designs (Bougioukas et al., 2023). In contrast, overviews involving interventions primarily assess the effectiveness and safety of interventions using SRs of RCTs, occasionally incorporating observational studies for rare adverse events
Items to consider before conducting an umbrella review	Aromataris et al. (2015) Baker et al. (2014)	There should be a plan to handle pooled estimates from different study designs, ensuring the methodology and study selection align with the umbrella review's primary objective (Aromataris et al., 2015; Baker et al., 2014)
Specifying the eligibility criteria for the umbrella review	Belbasis et al. (2022)	Set strict and precise criteria to exclude ineligible primary studies or if this is not feasible, examine each study captured by the SR/MA and recalculate the pooled estimates (see section on collecting, analyzing, and presenting outcome data) (Belbasis et al., 2022)
Conducting and reporting searches for systematic reviews	Fusar‐Poli and Radua (2018)	The literature search process should strictly adhere to PRISMA recommendations to ensure comprehensive and transparent reporting (Fusar‐Poli and Radua, 2018). Incorporate specific guidelines tailored to the types of studies being reviewed, such as applying the MOOSE guidelines for observational studies
Collecting, analyzing, and presenting outcome data	Fusar‐Poli and Radua (2018) Gianfredi et al. (2022)	Using various effect size measures for observational studies is beneficial, but employing a common effect size facilitates easier comparisons (Fusar‐Poli and Radua, 2018). To establish temporality and minimize reverse causation, sensitivity analysis including only prospective studies is advised, while acknowledging that MAs of observational studies often face biases affecting sample representativeness (Fusar‐Poli and Radua, 2018; Gianfredi et al., 2022)
Collecting and presenting data on the methodological quality (risk of bias) of primary studies contained within the included systematic reviews	Belbasis et al. (2022) Gianfredi et al. (2022)	When some SRs and MAs include risk‐of‐bias assessments using standardized tools, researchers are encouraged to summarize these assessments in a clear tabular format (Belbasis et al., 2022). The inherent heterogeneity in exposure and outcome assessment from original studies often persists in MAs and umbrella reviews, posing challenges for reliable conclusions. To address this, establish and adhere to strict inclusion and exclusion criteria for SRs and MAs (Gianfredi et al., 2022)
Assessing the methodological quality and certainty of the included systematic reviews	Arango et al. (2021) Janiaud et al. (2021)	The certainty of evidence from SRs of observational studies should be assessed with quantitative criteria (i.e., GRADE), including sensitivity analyses for temporality, and findings should be discussed with input from content experts. (Arango et al., 2021; Janiaud et al., 2021)

### What types of questions can be answered using the umbrella review format?

Bougioukas and colleagues (Bougioukas et al., 2023) stated that umbrella reviews of observational studies typically examine the epidemiological link between exposures (both genetic and non‐genetic) and disease development using data from SRs. These reviews often investigate potential risk or protective factors, but can also measure biomarkers, including SRs of different observational study designs (e.g., cross‐sectional, case‐control, and cohort studies). These associations often cannot be examined through experimental studies, and only observational evidence exists. In contrast, overviews of SRs on interventions evaluate the effectiveness and safety of one or more interventions for disease prevention or treatment and primarily use data from SRs of RCTs, while only occasionally using data from observational studies for rare adverse events. For example, some umbrella reviews on the safety of interventions can include SRs of RCTs and cohort or case‐control studies, examining them separately, using observational studies for events that are rare or require long follow‐up to be detected. Other SRs can focus on rates of medical conditions or markers of given conditions or outcomes, and in these cases, SRs of cross‐sectional studies are typically included.

### Items to consider before conducting an umbrella review

Two documents reported the importance of determining the inclusion criteria for SRs a priori (Aromataris et al., 2015; Baker et al., 2014). The methodology and study selection should align with the primary objective of the umbrella review, and all the steps of the umbrella review need to account for the type of eligible studies (Aromataris et al., 2015; Baker et al., 2014). Given that umbrella reviews vary in quality and might at times merge results from different study designs, a plan on how to handle pooled estimates from diverse study designs, particularly whether and why observational studies assessing epidemiological associations should or should not be combined, should be reported.

### Specifying the eligibility criteria for the umbrella review

Belbasis and colleagues (Belbasis et al., 2022) cautioned that SRs and MAs may have different eligibility criteria than those intended in the umbrella review. SRs might use similar definitions for different constructs. For instance, SRs might claim to focus on mental disorders but might have included reporting on subjects with mental health symptoms but no formal diagnosis (i.e. using the Diagnostic and Statistical Manual of Mental Disorders or International Classification of Diseases). Another example is that SRs might claim to include longitudinal studies, but also include cross‐sectional studies. In such cases, it is crucial to set strict and precise criteria to exclude SRs that report on studies that do not meet the inclusion criteria. Authors can alternatively examine each study captured by the SR/MA and re‐calculate the pooled estimates, focusing on evidence that is eligible for and applicable to the umbrella review (see section on collecting, analyzing, and presenting outcome data).

### Conducting and reporting searches for systematic reviews

Fusar‐Poli and colleagues (Fusar‐Poli and Radua, 2018) suggested that the literature search process should strictly adhere to the PRISMA recommendations to ensure comprehensive and transparent reporting. In addition to following the PRISMA guidelines concerning searching, it is also important to incorporate specific guidelines tailored to the types of studies being reviewed. For instance, when dealing with observational studies, the Meta‐analysis of Observational Studies in Epidemiology (MOOSE) guidelines (Stroup et al., 2000) should be applied. These recommendations were formulated to guide reporting in SRs and MAs before the PRIOR checklist was introduced and indirectly called for specific reporting guidance for umbrella reviews of observational studies. There was no guidance specifically on conducting database or gray literature searches.

### Collecting, analyzing, and presenting outcome data

Using various effect size measures for observational studies can be beneficial, as each measure is appropriate for specific study types. However, employing a common effect size across all factors allows for easier comparisons (Fusar‐Poli and Radua, 2018). To establish the temporality of association and minimize reverse causation, temporality should be addressed through a sensitivity analysis that includes only prospective studies (Fusar‐Poli and Radua, 2018). Gianfredi and colleagues discussed that MAs of observational studies are often affected by potential selection bias or lost‐to‐follow‐up bias, which can undermine sample representativeness (Gianfredi et al., 2022). Additionally, heterogeneity in confounding variables, small sample sizes, or inadequate power are potential concerns. Prioritizing adjusted estimates over unadjusted ones is important since different adjustments can lead to varying results and inferences. Where possible, standardization of outcomes, and their operationalization and reporting, may be beneficial.

### Collecting and presenting data on the methodological quality (risk of bias) of primary studies contained within the included systematic reviews

When some SRs and MAs include risk‐of‐bias assessments using standardized tools, such as the JBI tool for observational studies (Moola et al., 2024) and the Risk Of Bias In Non‐randomized Studies ‐ of Exposures (ROBINS‐E) tool (Higgins et al., 2024), researchers are encouraged to summarize these assessments in a clear and organized tabular format (Belbasis et al., 2022). Often, the inherent heterogeneity in exposure and outcome assessment found in the original studies carries over into the MAs and subsequently into the umbrella reviews. This variability can pose significant challenges in drawing reliable conclusions. To address this issue effectively, it is recommended to establish and adhere to a strict definition of inclusion and exclusion criteria for the SRs and MAs included in the umbrella review (Gianfredi et al., 2022).

### Assessing the methodological quality of the included systematic reviews and the certainty of estimates reported by the included systematic reviews

The certainty of evidence from SRs of observational studies should be assessed, ideally with quantitative criteria and with sensitivity analyses accounting for the temporality of putative risk or protective factors. The Grading of Recommendations Assessment, Development and Evaluation (GRADE) tool (Guyatt et al., 2008) can be used to assess and report the certainty of evidence for each clinically important outcome. If possible, authors should extract and report GRADE assessments from included SRs, but they may need to conduct their own assessments if the included SRs lack comprehensive GRADE evaluations or use different tools. A robust set of quantitative criteria has been largely used across umbrella reviews of observational studies (Arango et al., 2021; Janiaud et al., 2021; Solmi et al., 2023). These criteria use information on the amount of evidence (e.g., >1000 cases or more than >20,000 cumulative participants for continuous outcomes), statistical strength of the evidence (e.g., *p*‐value, using cut‐offs of <10‐6 and <10‐3), consistency (e.g., not large between‐study heterogeneity, 95% predictive interval excluding the null), and no obvious bias in small‐study effect and excess significance tests. The clinical or public health interpretation of umbrella review findings should be discussed in the context of content expertise among the authors of the umbrella review.

## DISCUSSION

This scoping review identified eight articles, which revealed that some guidance already exists for the conduct and reporting of umbrella reviews of observational studies, but it is scattered across different articles. There are still some gaps or uncertainties in the recommendations for various stages of conducting and/or reporting an umbrella review, namely: (1) choosing between conducting an umbrella review of observational studies and a new SR, (2) author team composition and roles, (3) target audience of the umbrella review of observational studies, (4) developing and registering an umbrella review of observational studies' protocol, (5) collecting and presenting data on descriptive characteristics of included SRs (and their primary studies), (6) consideration and management of overlapping SRs, (7) interpreting outcome data and drawing conclusions, and (8) updating the umbrella review. At the same time, aspects to account for specifically in umbrella reviews of observational studies have emerged that call for dedicated and specific reporting guidance for this study design. For instance, the variability of temporal sequences of exposure and outcomes across different study designs and the differences in quality and certainty assessments in SRs of observational studies compared to RCTs (Solmi et al., 2018a). Our findings are corroborated by a recent scoping review, which identified inconsistencies in the certainty of evidence assessments among umbrella reviews of observational studies published between 2010 and 2021 (Sadoyu et al., 2022). Our review of existing guidance documents on the conduct and/or reporting of umbrella reviews of observational studies sets the ground for Step 3 of the PRIUR‐CCC project, that is, the Delphi survey, which will include three rounds of expert participant surveys followed by response analysis. These results will inform the creation of the final PRIUR‐CCC statement, which will include formal reporting guidelines for umbrella reviews of observational studies on epidemiological associations (Solmi et al., 2024).

We employed a transparent and rigorous approach to compile information from all available guidance documents applicable to umbrella reviews of observational studies on epidemiological associations. We adapted the search strategies used in the PRIOR scoping review on guidance for overviews of reviews of healthcare interventions (Gates et al., 2020), which included comprehensive database searches and manual searches of various sources. Notably, the guidance summarized in our review may not be directly applicable to other types of umbrella reviews, such as those focusing on diagnostic accuracy, experimental studies, or qualitative research.

The title/abstract and full‐text screening of the records and the extraction of recommendations from them were conducted independently and in duplicate, serving as a strength of the study. The inclusion criterion of records regardless of language avoids potentially overlooking relevant information. Furthermore, we screened a wide range of literature, including journals and gray literature sources, to ensure a thorough review of the recommendations. However, while we explored both publicly available search results and those accessible through our university library systems and interlibrary loans, we acknowledge that there still exists a possibility that not all relevant recommendations for the conduct and reporting of umbrella reviews of observational studies were captured.

In conclusion, the growing popularity of umbrella reviews has resulted in the creation of reporting guidance on such reviews of healthcare interventions (Gates et al., 2022). In the case of umbrella reviews for observational studies on epidemiological associations, specifically, there is scattered guidance within the current literature, leaving several areas of uncertainty. The findings of this scoping review will contribute to the creation of an evidence‐based and consensus‐driven guideline, the PRIOR‐extension for Umbrella Reviews of Cross‐sectional, Case‐control, and Cohort studies (PRIUR‐CCC). The development of this reporting guideline adheres to established EQUATOR principles and builds upon the foundational PRIOR statement (Gates et al., 2022), ultimately providing a standardized framework to improve the quality and consistency of reporting.

## AUTHOR CONTRIBUTIONS

**Carl Zhou**: Conceptualization; formal analysis; writing—original draft; writing—review and editing. **Nicholas Fabiano**: Data curation; formal analysis; writing—review and editing. **Arnav Gupta**: Data curation; formal analysis; writing—review and editing. **Stanley Wong**: Data curation; formal analysis; writing—review and editing. **Kelly D. Cobey**: Conceptualization; writing—review and editing. **David Moher**: Conceptualization; writing—review and editing. **Sanam Ebrahimzadeh**: Conceptualization; writing—review and editing. **Jeremy Y. Ng**: Conceptualization; writing—review and editing. **Elena Dragioti**: Conceptualization; writing—review and editing. **Jae Il Shin**: Conceptualization; writing—review and editing. **Joaquim Radua**: Conceptualization; writing—review and editing. **Samuele Cortese**: Conceptualization; writing—review and editing. **Beverley Shea**: Conceptualization; writing—review and editing. **Nicola Veronese**: Conceptualization; writing—review and editing. **Lisa Hartling**: Conceptualization; writing—review and editing. **Michelle Pollock**: Conceptualization; writing—review and editing. **Stefania Papatheodorou**: Conceptualization; writing—review and editing. **John P. A. Ioannidis**: Conceptualization; writing—review and editing. **Marco Solmi**: Conceptualization; project administration; supervision; writing—original draft; writing—review and editing.

## CONFLICT OF INTEREST STATEMENT

Samuele Cortese has declared reimbursement for travel and accommodation expenses from the Association for Child and Adolescent Central Health (ACAMH) in relation to lectures delivered for ACAMH, the Canadian AADHD Alliance Resource, the British Association of Psychopharmacology, Healthcare Convention and CCM Group team for educational activity on ADHD, and has received honoraria from Medice. Marco Solmi received honoraria/has been a consultant for Angelini, AbbVie, Boehringer Ingelheim, Lundbeck, Otsuka.

## ETHICAL CONSIDERATIONS

The broader PRIUR‐CCC project, for which this study is a part, has been approved by The Ottawa Health Science Network Research Ethics Board (20220639‐01H).

## Supporting information

Supporting Information S1

## Data Availability

The data used in this manuscript are directly reported in the manuscript itself or included as a Supporting Information S1.
