# Supplementary material for: Guidance for umbrella reviews of observational studies: A scoping review
Source: JCPP Adv. 2025 Aug 1;6(1):e70017. doi: 10.1002/jcv2.70017 (PMC12973145; doi:10.1002/jcv2.70017)
Supplement: Supplementary file 1 — Supporting Information S1 [file JCV2-6-e70017-s001.docx]

**Supporting Information**

**Guidance for umbrella reviews of observational studies: a scoping review**

Carl Zhou^1^, Nicholas Fabiano^1^, Arnav Gupta^2,3^, Stanley Wong^4^, Kelly D. Cobey^5,6^, David Moher^5^, Sanam Ebrahimzadeh^7^, Jeremy Y. Ng^8^, Elena Dragioti^9^, Jae Il Shin^10,11^, Joaquim Radua^12-14^, Samuele Cortese^15-19^, Beverley Shea^5,6^, Nicola Veronese^20^, Lisa Hartling^21^, Michelle Pollock^22^, Stefania Papatheodorou^23,24^, John P.A. Ioannidis^25-27^, Marco Solmi^1,5,7,28,29^

1. Department of Psychiatry, University of Ottawa, Ottawa, Canada
2. Department of Medicine, University of Calgary, Calgary, Canada
3. College of Public Health, Kent State University, Kent, USA
4. Department of Psychiatry, University of Toronto, Toronto, Canada
5. School of Epidemiology and Public Health, Faculty of Medicine, University of Ottawa, Ottawa, Canada
6. University of Ottawa Heart Institute, Ottawa, Canada
7. Ottawa Hospital Research Institute, Ottawa, Canada
8. Centre for Journalology, Ottawa Hospital Research Institute, Ottawa, Canada
9. Research Laboratory Psychology of Patients, Families, and Health Professionals, Department of Nursing, School of Health Sciences, University of Ioannina, Ioannina, Greece
10. Department of Pediatrics, Yonsei University College of Medicine, Seoul, South Korea
11. Severance Underwood Meta-Research Center, Institute of Convergence Science, Yonsei University, Seoul, South Korea
12. Imaging of Mood and Anxiety Related Disorders (IMARD) Group, d'Investigacions Biomèdiques August Pi i Sunyer (IDIBAPS), CIBERSAM ES, Barcelona, Spain
13. Department of Psychosis Studies, Institute of Psychiatry, Psychology and Neuroscience, King's College London, London, UK
14. Department of Medicine, University of Barcelona, Barcelona, Spain
15. Developmental EPI (Evidence synthesis, Prediction, Implementation) Lab, Centre for Innovation in Mental Health, School of Psychology, Faculty of Environmental and Life Sciences, University of Southampton, Southampton, UK
16. Clinical and Experimental Sciences (CNS and Psychiatry), Faculty of Medicine, University of Southampton, Southampton, UK
17. Hampshire and Isle of Wight Healthcare, NHS Foundation Trust, Southampton, UK
18. Hassenfeld Children's Hospital at NYU Langone, New York University Child Study Center, New York City, USA
19. DiMePRe-J-Department of Precision and Rigenerative Medicine-Jonic Area, University of Bari "Aldo Moro", Bari, Italy
20. Geriatric Unit, Department of Internal Medicine and Geriatrics, University of Palermo, Palermo, Italy
21. Alberta Research Centre for Health Evidence, Department Pediatrics, Faculty of Medicine & Dentistry, University of Alberta, Edmonton, Canada
22. Institute of Health Economics, Edmonton, Canada
23. Department of Biostatistics and Epidemiology, Rutgers School of Public Health, Piscataway, USA
24. Department of Epidemiology, Harvard TH Chan School of Public Health, Boston, USA
25. Department of Medicine, Stanford University, Stanford, USA
26. Department of Epidemiology and Population Health, Stanford University, Stanford, USA
27. Meta-Research Innovation Center at Stanford (METRICS), Stanford University, Stanford, USA
28. Department of Mental Health, The Ottawa Hospital, Ottawa, Canada
29. Department of Child and Adolescent Psychiatry, Charité Universitätsmedizin, Berlin, Germany

Marco Solmi (Corresponding author): [msolmi@toh.ca](mailto:msolmi@toh.ca), <https://orcid.org/0000-0003-4877-7233>

**Authors’ contributions**: NF, AG, SW contributed to screening, data extraction, and verification. NF, AG, SW, CZ contributed to data analysis. CZ, MS drafted the first version of the manuscript. All authors contributed to the protocol, edited, and approved the current version of the manuscript. MS oversaw all aspects of the project.

**Funding**: None

**Availability of data and materials**: The data used in this manuscript are directly reported in the manuscript itself or included as a supplementary file.

**Ethics approval and consent to participate**: The broader PRIUR-CCC project, for which this study is a part, has been approved by The Ottawa Health Science Network Research Ethics Board (20220639-01H).

**Competing interests**: Samuele Cortese has declared reimbursement for travel and accommodation expenses from the Association for Child and Adolescent Central Health (ACAMH) in relation to lectures delivered for ACAMH, the Canadian AADHD Alliance Resource, the British Association of Psychopharmacology, Healthcare Convention and CCM Group team for educational activity on ADHD, and has received honoraria from Medice. Marco Solmi received honoraria/has been a consultant for Angelini, AbbVie, Boehringer Ingelheim, Lundbeck, Otsuka.

**Acknowledgements**: Samuele Cortese, NIHR Research Professor (NIHR303122) is funded by the NIHR for this research project. The views expressed in this publication are those of the author(s) and not necessarily those of the NIHR, NHS or the UK Department of Health and Social Care. Samuele Cortese is also supported by NIHR grants NIHR203684, NIHR203035, NIHR130077, NIHR128472, RP-PG-0618-20003 and by grant 101095568-HORIZONHLTH- 2022-DISEASE-07-03 from the European Research Executive Agency. Lisa Hartling is supported by a Canada Research Chair in Knowledge Synthesis and Translation.

**Table S1.** Completed Preferred Reporting Items for Systematic reviews and Meta-Analyses extension for Scoping Reviews (PRISMA-ScR) Checklist.

| **SECTION** | **ITEM** | **PRISMA-ScR CHECKLIST ITEM** | **REPORTED SECTION** |
| --- | --- | --- | --- |
| **TITLE** | | | |
| Title | 1 | Identify the report as a scoping review. | Title page |
| **ABSTRACT** | | | |
| Structured summary | 2 | Provide a structured summary that includes (as applicable): background, objectives, eligibility criteria, sources of evidence, charting methods, results, and conclusions that relate to the review questions and objectives. | Abstract |
| **INTRODUCTION** | | | |
| Rationale | 3 | Describe the rationale for the review in the context of what is already known. Explain why the review questions/objectives lend themselves to a scoping review approach. | Introduction |
| Objectives | 4 | Provide an explicit statement of the questions and objectives being addressed with reference to their key elements (e.g., population or participants, concepts, and context) or other relevant key elements used to conceptualize the review questions and/or objectives. | Introduction |
| **METHODS** | | | |
| Protocol and registration | 5 | Indicate whether a review protocol exists; state if and where it can be accessed (e.g., a Web address); and if available, provide registration information, including the registration number. | Methods |
| Eligibility criteria | 6 | Specify characteristics of the sources of evidence used as eligibility criteria (e.g., years considered, language, and publication status), and provide a rationale. | Methods |
| Information sources* | 7 | Describe all information sources in the search (e.g., databases with dates of coverage and contact with authors to identify additional sources), as well as the date the most recent search was executed. | Methods, eTable 2 |
| Search | 8 | Present the full electronic search strategy for at least 1 database, including any limits used, such that it could be repeated. | eTable 2 |
| Selection of sources of evidence† | 9 | State the process for selecting sources of evidence (i.e., screening and eligibility) included in the scoping review. | Methods |
| Data charting process‡ | 10 | Describe the methods of charting data from the included sources of evidence (e.g., calibrated forms or forms that have been tested by the team before their use, and whether data charting was done independently or in duplicate) and any processes for obtaining and confirming data from investigators. | Methods |
| Data items | 11 | List and define all variables for which data were sought and any assumptions and simplifications made. | Methods |
| Critical appraisal of individual sources of evidence§ | 12 | If done, provide a rationale for conducting a critical appraisal of included sources of evidence; describe the methods used and how this information was used in any data synthesis (if appropriate). | N/A |
| Synthesis of results | 13 | Describe the methods of handling and summarizing the data that were charted. | Methods |
| **RESULTS** | | | |
| Selection of sources of evidence | 14 | Give numbers of sources of evidence screened, assessed for eligibility, and included in the review, with reasons for exclusions at each stage, ideally using a flow diagram. | Results |
| Characteristics of sources of evidence | 15 | For each source of evidence, present characteristics for which data were charted and provide the citations. | Results, eTable 2, eFiles 1 and 2 |
| Critical appraisal within sources of evidence | 16 | If done, present data on critical appraisal of included sources of evidence (see item 12). | N/A |
| Results of individual sources of evidence | 17 | For each included source of evidence, present the relevant data that were charted that relate to the review questions and objectives. | Results, Tables 1 and 2 |
| Synthesis of results | 18 | Summarize and/or present the charting results as they relate to the review questions and objectives. | Results, Tables 1 and 2 |
| **DISCUSSION** | | | |
| Summary of evidence | 19 | Summarize the main results (including an overview of concepts, themes, and types of evidence available), link to the review questions and objectives, and consider the relevance to key groups. | Discussion, Tables 1 and 2 |
| Limitations | 20 | Discuss the limitations of the scoping review process. | Discussion |
| Conclusions | 21 | Provide a general interpretation of the results with respect to the review questions and objectives, as well as potential implications and/or next steps. | Discussion |
| **FUNDING** | | | |
| Funding | 22 | Describe sources of funding for the included sources of evidence, as well as sources of funding for the scoping review. Describe the role of the funders of the scoping review. | Title page |

JBI = Joanna Briggs Institute; PRISMA-ScR = Preferred Reporting Items for Systematic reviews and Meta-Analyses extension for Scoping Reviews.

* Where *sources of evidence* (see second footnote) are compiled from, such as bibliographic databases, social media platforms, and Web sites.

† A more inclusive/heterogeneous term used to account for the different types of evidence or data sources (e.g., quantitative and/or qualitative research, expert opinion, and policy documents) that may be eligible in a scoping review as opposed to only studies. This is not to be confused with *information sources* (see first footnote).

‡ The frameworks by Arksey and O’Malley (6) and Levac and colleagues (7) and the JBI guidance (4, 5) refer to the process of data extraction in a scoping review as data charting*.*

§ The process of systematically examining research evidence to assess its validity, results, and relevance before using it to inform a decision. This term is used for items 12 and 19 instead of "risk of bias" (which is more applicable to systematic reviews of interventions) to include and acknowledge the various sources of evidence that may be used in a scoping review (e.g., quantitative and/or qualitative research, expert opinion, and policy document).

*From:* Tricco AC, Lillie E, Zarin W, O'Brien KK, Colquhoun H, Levac D, et al. PRISMA Extension for Scoping Reviews (PRISMAScR): Checklist and Explanation. Ann Intern Med. 2018;169:467–473. [doi: 10.7326/M18-0850](http://annals.org/aim/fullarticle/2700389/prisma-extension-scoping-reviews-prisma-scr-checklist-explanation).**Table S2. Details of the electronic search strategy.**

**ELECTRONIC SEARCH STRATEGIES**

**1.** **Reference Tracking**

**Start Date:** 2020 (inclusive)

**End Date:** December 22, 2024

**Procedure:** For each target article, we searched for "citing" references (Scopus), "cited" references (reference lists), and "similar articles" (PubMed).

| **Target articles (n=46)** | **Similar articles (PubMed)** | **Citing references (Scopus)** |
| --- | --- | --- |
| Aromataris E, Fernandez R, Godfrey CM, Holly C, Khalil H, Tungpunkom P. Summarizing systematic reviews: methodological development, conduct and reporting of an umbrella review approach. International Journal of Evidence-Based Healthcare. 2015;13(3):132-40. PMID: 26360830 | 683 | 1270 |
| Aromataris E, Fernandez RS, Godfrey C, Holly C, Khalil H, Tungpunkom P. Methodology for JBI umbrella reviews. In: The Joanna Briggs Institute Reviewers Manual 2014. Adelaide, Australia: The Joanna Briggs Institute; 2014. | 0 | 0 |
| Baker PRA, Costello JT, Dobbins M, B. Waters E. The benefits and challenges of conducting an overview of systematic reviews in public health: a focus on physical activity. Journal of Public Health. 2014;36(3):517-21. PMID: 25085438 | 6 | 14 |
| Ballard M, Montgomery P. Risk of bias in overviews of reviews: A scoping review of methodological guidance and four-item checklist. Research Synthesis Methods. 2017;8(1):92-108. PMID: 28074553 | 73 | 57 |
| Becker LA, Oxman AD. Chapter 22: Overviews of reviews. In: Higgins JPT, Green S (editors). Cochrane handbook for systematic reviews of interventions (version 5.1.0). London, UK: The Cochrane Collaboration; 2011. | 0 | 0 |
| Bougioukas KI, Bouras E, Apostolidou-Kiouti F, Kokkali S, Arvanitidou M, Haidich AB. Reporting guidelines on how to write a complete and transparent abstract for overviews of systematic reviews of health care interventions. Journal of Clinical Epidemiology. 2019;106:70-9. PMID: 30336211 | 65 | 44 |
| Bougioukas KI, Liakos A, Tsapas A, Ntzani E, Haidich AB. Preferred reporting items for overviews of systematic reviews including harms checklist: A pilot tool to be used for balanced reporting of benefits and harms. Journal of clinical epidemiology. 2018;93:9-24. PIMD: 29037888 | 111 | 160 |
| Buchter RB, Pieper D. Most overviews of Cochrane reviews neglected potential biases from dual authorship. Journal of Clinical Epidemiology. 2016;77:91-4. PMID: 27131430 | 44 | 2 |
| Caird J, Sutcliffe K, Kwan I, Dickson K, Thomas J. Mediating policy-relevant evidence at speed: Are systematic reviews of systematic reviews a useful approach? Evidence & Policy. 2015;11(1):81-97. | 0 | 10 |
| Caldwell DM, Welton NJ, Ades AE. Mixed treatment comparison analysis provides internally coherent treatment effect estimates based on overviews of reviews and can reveal inconsistency. Journal of Clinical Epidemiology. 2010;63(8):875-82. PMID: 20080027 | 21 | 30 |
| Conn VS, Coon Sells TG. WJNR welcomes umbrella reviews. Los Angeles, CA: Sage Publications; 2014. PMID: 24391147 | 3 | 3 |
| Cooper H, Koenka AC. The overview of reviews: unique challenges and opportunities when research syntheses are the principal elements of new integrative scholarship. The American Psychologist. 2012;67(6):446-62. PMID: 22352742 | 7 | 0 |
| Crick K, Wingert A, Williams K, Fernandes RM, Thomson D, Hartling L. An evaluation of harvest plots to display results of meta-analyses in overviews of reviews: A cross-sectional study. BMC Medical Research Methodology. 2015;15(1):91. PMID: 26502717 | 22 | 2 |
| Elliott L, Crombie IK, Irvine L, Cantrell J, Taylor J. The effectiveness of public health nursing: the problems and solutions in carrying out a review of systematic reviews. Journal of Advanced Nursing. 2004;45(2):117-25. PMID: 14705995 | 11 | 0 |
| Esposito M. Editorial: Overviews and umbrella reviews. European Journal of Oral Implantology. 2018;11(3):255. PIMD: 30246180 | 12 | 0 |
| Faggion CM, Jr., Cavero KD. Overview authors rarely defined systematic reviews that are included in their overviews. Journal of Clinical Epidemiology. 2019. S0895-4356(18)30640-1. PMID: 30684566 | 70 | 9 |
| Fusar-Poli P, Radua J. Ten simple rules for conducting umbrella reviews. Evidence-based Mental Health. 2018;21(3):95-100. PMID: 30006442 | 82 | 360 |
| Hartling L, Chisholm A, Thomson D, Dryden DM. A descriptive analysis of overviews of reviews published between 2000 and 2011. PloS One. 2012;7(11):e49667. PMID: 23166744 | 42 | 79 |
| Hartling L, Vandermeer B, Fernandes RM. Systematic reviews, overviews of reviews and comparative effectiveness reviews: A discussion of approaches to knowledge synthesis. Evidence-based Child Health: a Cochrane Review Journal. 2014;9(2):486-94. PMID: 25404611 | 48 | 21 |
| Hemming K, Bowater RJ, Lilford RJ. Pooling systematic reviews of systematic reviews: A Bayesian panoramic meta‐analysis. Statistics in Medicine. 2012;31(3):201-16. PMID: 21965138 | 4 | 4 |
| Hunt H, Pollock A, Campbell P, Estcourt L, Brunton G. An introduction to overviews of reviews: planning a relevant research question and objective for an overview. Systematic Reviews. 2018;7(1):39. PMID: 29490699 | 82 | 187 |
| Ioannidis J. Next-generation systematic reviews: prospective meta-analysis, individual-level data, networks and umbrella reviews. British Journal of Sports Medicine. 2017;51(20):1456-8. PMID: 28223307 | 10 | 113 |
| Li L, Tian J, Tian H, Sun R, Liu Y, Yang K. Quality and transparency of overviews of systematic reviews. Journal of Evidence-based Medicine. 2012;5(3):166-73. PMID: 23672223 | 35 | 26 |
| Lunny C, Brennan SE, McDonald S, McKenzie JE. Toward a comprehensive evidence map of overview of systematic review methods: Paper 1-purpose, eligibility, search and data extraction. Systematic Reviews. 2017;6(1):231. PMID: 29162130 | 97 | 88 |
| Lunny C, Brennan SE, McDonald S, McKenzie JE. Toward a comprehensive evidence map of overview of systematic review methods: Paper 2-risk of bias assessment; synthesis, presentation and summary of the findings; and assessment of the certainty of the evidence. Systematic Reviews. 2018;7(1):159. PMID: 30314530 | 256 | 80 |
| McKenzie JE, Brennan SE. Overviews of systematic reviews: great promise, greater challenge. Systematic Reviews. 2017;6(1):185. PMID: 28886726 | 38 | 65 |
| Pieper D, Antoine SL, Morfeld JC, Mathes T, Eikermann M. Methodological approaches in conducting overviews: Current state in HTA agencies. Research Synthesis Methods. 2014;5(3):187-99. PMID: 26052845 | 52 | 14 |
| Pieper D, Antoine S-L, Mathes T, Neugebauer EA, Eikermann M. Systematic review finds overlapping reviews were not mentioned in every other overview. Journal of Clinical Epidemiology. 2014;67(4):368-75. PMID: 24581293 | 33 | 497 |
| Pieper D, Antoine S-L, Neugebauer EA, Eikermann M. Up-to-dateness of reviews is often neglected in overviews: A systematic review. Journal of Clinical Epidemiology. 2014;67(12):1302-8. PMID: 25281222 | 32 | 28 |
| Pieper D, Buchter RB, Antoine SL, Eikermann M. [Overviews - status quo, potentials and perspectives]. Zeitschrift fur Evidenz, Fortbildung und Qualitat im Gesundheitswesen. 2013;107(9-10):592-6. PMID: 24315329 | 29 | 0 |
| Pieper D, Buechter R, Jerinic P, Eikermann M. Overviews of reviews often have limited rigor: A systematic review. Journal of Clinical Epidemiology. 2012;65(12):1267-73. PMID: 22959594 | 54 | 70 |
| Pieper D, Pollock M, Fernandes RM, Buchter RB, Hartling L. Epidemiology and reporting characteristics of overviews of reviews of healthcare interventions published 2012-2016: Protocol for a systematic review. Systematic Reviews. 2017;6(1):73. PMID: 28388960 | 76 | 10 |
| Pieper D, Waltering A, Holstiege J, Buchter RB. Quality ratings of reviews in overviews: A comparison of reviews with and without dual (co-)authorship. Systematic Reviews. 2018;7(1):63. PMID: 29690911 | 57 | 5 |
| Piso B, Semlitsch T, Reinsperger I, Breuer J, Kaminski-Hartenthaler A, Kien C, et al. [Practical experience with overviews of reviews--valuable decision aid or academic exercise?]. Zeitschrift fur Evidenz, Fortbildung und Qualitat im Gesundheitswesen. 2015;109(4-5):300-8. PMID: 26354130 | 23 | 0 |
| Pollock A, Campbell P, Brunton G, Hunt H, Estcourt L. Selecting and implementing overview methods: implications from five exemplar overviews. Systematic Reviews. 2017;6(1):145. PMID: 28720141 | 41 | 63 |
| Pollock M, Fernandes RM, Becker LA, Featherstone R, Hartling L. What guidance is available for researchers conducting overviews of reviews of healthcare interventions? A scoping review and qualitative metasummary. Systematic Reviews. 2016;5(1):190. PMID: 27842604 | 404 | 96 |
| Pollock M, Fernandes RM, Hartling L. Evaluation of AMSTAR to assess the methodological quality of systematic reviews in overviews of reviews of healthcare interventions. BMC Medical Research Methodology. 2017;17(1):48. PMID: 28335734 | 395 | 58 |
| Pollock M, Fernandes RM, Newton AS, Scott SD, Hartling L. The impact of different inclusion decisions on the comprehensiveness and complexity of overviews of reviews of healthcare interventions. Systematic Reviews. 2019;8(1):18. PMID: 30635048 | 133 | 24 |
| Pollock M, Fernandes RM, Newton AS, Scott SD, Hartling L. A decision tool to help researchers make decisions about including systematic reviews in overviews of reviews of healthcare interventions. Systematic Reviews. 2019;8(1):29. PMID: 30670086 | 187 | 62 |
| Ryan RE, Kaufman CA, Hill SJ. Building blocks for meta-synthesis: data integration tables for summarising, mapping, and synthesising evidence on interventions for communicating with health consumers. BMC Medical Research Methodology. 2009;9(1):16. PMID: 19261177 | 15 | 8 |
| Schultz A, Goertzen L, Rothney J, Wener P, Enns J, Halas G, et al. A scoping approach to systematically review published reviews: Adaptations and recommendations. Research Synthesis Methods. 2018;9(1):116-23. PMID: 29032590 | 67 | 34 |
| Silva V, Grande AJ, Carvalho AP, Martimbianco AL, Riera R. Overview of systematic reviews - a new type of study. Part II. Sao Paulo Medical Journal. 2015;133(3):206-17. PMID: 25388685 | 3 | 18 |
| Silva V, Grande AJ, Martimbianco AL, Riera R, Carvalho AP. Overview of systematic reviews - a new type of study: part I: why and for whom? Sao Paulo Medical Journal. 2012;130(6):398-404. PMID: 23338737 | 4 | 13 |
| Smith V, Devane D, Begley CM, Clarke M. Methodology in conducting a systematic review of systematic reviews of healthcare interventions. BMC Medical Research Methodology. 2011;11(1):15. PMID: 21291558 | 31 | 487 |
| Thomson D, Foisy M, Oleszczuk M, Wingert A, Chisholm A, Hartling L. Overview of reviews in child health: evidence synthesis and the knowledge base for a specific population. Evidence‐Based Child Health: A Cochrane Review Journal. 2013;8(1):3-10. PMID: 23878121 | 40 | 3 |
| Thomson D, Russell K, Becker L, Klassen T, Hartling L. The evolution of a new publication type: Steps and challenges of producing overviews of reviews. Research Synthesis Methods. 2010;1(3‐4):198-211. PMID: 26061466 | 30 | 6 |

**Appendix S1.** Documents excluded following full-text review.

**Documents excluded following full-text review (n=88).**

**Not observational studies (n=35)**

Pollock M, Fernandes RM, Becker LA, Pieper D, Hartling L. Chapter V: Overviews of Reviews. In: Higgins JPT, Thomas J, Chandler J, Cumpston M, Li T, Page MJ, Welch VA (editors). Cochrane Handbook for Systematic Reviews of Interventions version 6.4 (updated August 2023). Cochrane, 2023. Available from: www.training.cochrane.org/handbook.

Kho ME, Poitras VJ, Janssen I, et al. Development and application of an outcome-centric approach for conducting overviews of reviews. *Appl Physiol Nutr Metab*. 2020;45(10 (Suppl. 2)):S151-S164. doi:10.1139/apnm-2020-0564

López-López JA, Rubio-Aparicio M, Sánchez-Meca J. Overviews of Reviews: Concept and Development. *Psicothema*. 2022;34(2):175-181. doi:10.7334/psicothema2021.586

Gosling CJ, Solanes A, Fusar-Poli P, Radua J. metaumbrella: the first comprehensive suite to perform data analysis in umbrella reviews with stratification of the evidence. *BMJ Ment Health*. 2023;26(1):e300534. doi:10.1136/bmjment-2022-300534

Slim K, Marquillier T. Umbrella reviews: A new tool to synthesize scientific evidence in surgery. *J Visc Surg*. 2022;159(2):144-149. doi:10.1016/j.jviscsurg.2021.10.001

Choi GJ, Kang H. Introduction to Umbrella Reviews as a Useful Evidence-Based Practice. *J Lipid Atheroscler*. 2023;12(1):3-11. doi:10.12997/jla.2023.12.1.3

Rouleau G, Hong QN, Kaur N, Gagnon M, Côté J, Bouix-Picasso J, Pluye P. Systematic Reviews of Systematic Quantitative, Qualitative, and Mixed Studies Reviews in Healthcare Research: How to Assess the Methodological Quality of Included Reviews? *Journal of Mixed Methods Research*. 2023;17(1),51-69. doi:10.1177/15586898211054243

Shi X, Wallach JD. Umbrella reviews: a useful study design in need of standardisation *BMJ*. 2022;378:o1740. doi:10.1136/bmj.o1740

Belbasis L, Bellou V, Ioannidis JPA. Conducting umbrella reviews. *BMJ Medicine*. 2022;1:e000071. doi:10.1136/bmjmed-2021-000071

Bougioukas KI, Vounzoulaki E, Mantsiou CD, Savvides ED, Karakosta C, Diakonidis T, Tsapas A, Haidich AB. Methods for depicting overlap in overviews of systematic reviews: An introduction to static tabular and graphical displays. *J Clin Epidemiol*. 2021;132:34-45. doi:10.1016/j.jclinepi.2020.12.004

Franco JVA, Meza N. Authors should also report the support for judgment when applying AMSTAR 2. *J Clin Epidemiol*. 2021;138:240. doi:10.1016/j.jclinepi.2021.02.029

Pollock M, Fernandes RM, Hartling L. Evaluation of AMSTAR to assess the methodological quality of systematic reviews in overviews of reviews of healthcare interventions. *BMC Med Res Methodol*. 2017;17(1):48. Published 2017 Mar 23. doi:10.1186/s12874-017-0325-5

Pollock M, Fernandes RM, Newton AS, Scott SD, Hartling L. A decision tool to help researchers make decisions about including systematic reviews in overviews of reviews of healthcare interventions. *Syst Rev*. 2019;8(1):29. Published 2019 Jan 22. doi:10.1186/s13643-018-0768-8

Foisy M, Thomson D, Dryden DM, Fernandes R, Hartling L. Conducting overviews of reviews: lessons learned since 2006. 2014 Cochrane Colloquium, Hyderabad.

Hartling L, Fernandes R, Becker L, Foisy M. Comparing multiple treatments: an introduction to overviews of reviews. 2015 Cochrane Colloquium, Vienna.

Thomson D, Russell K, Becker L, Klassen T, Hartling L. The evolution of a new publication type: Steps and challenges of producing overviews of reviews. *Res Synth Methods*. 2010;1(3-4):198-211. doi:10.1002/jrsm.30

Caldwell DM, Welton NJ, Ades AE. Mixed treatment comparison analysis provides internally coherent treatment effect estimates based on overviews of reviews and can reveal inconsistency. *J Clin Epidemiol*. 2010;63(8):875-882. doi:10.1016/j.jclinepi.2009.08.025

Higgins JPT, Thomas J, Chandler J, Cumpston M, Li T, Page MJ, Welch VA (editors). Cochrane Handbook for Systematic Reviews of Interventions version 6.4 (updated August 2023). Cochrane, 2023. Available from www.training.cochrane.org/handbook.

Salanti G, Becker L, Caldwell D, Higgins J, Li T, Schmid C. Evolution of Cochrane Intervention Reviews and Overviews of Reviews to better accommodate comparisons among multiple interventions. Cochrane, 2011. Available from https://methods.cochrane.org/cmi/milan-report.

Caird J, Sutcliffe K, Kwan I, Dickson K, Thomas J. Mediating policy-relevant evidence at speed: are systematic reviews of systematic reviews a useful approach? *Evidence & Policy*. 2015;11(1):81-97. doi:10.1332/174426514X13988609036850

Papatheodorou SI, Evangelou E. Umbrella Reviews: What They Are and Why We Need Them. *Methods Mol Biol*. 2022;2345:135-146. doi:10.1007/978-1-0716-1566-9_8

Smith V, Devane D, Begley CM, Clarke M. Methodology in conducting a systematic review of systematic reviews of healthcare interventions. *BMC Med Res Methodol*. 2011;11(1):15. Published 2011 Feb 3. doi:10.1186/1471-2288-11-15

Hemming K, Bowater RJ, Lilford RJ. Pooling systematic reviews of systematic reviews: a Bayesian panoramic meta-analysis. *Stat Med*. 2012;31(3):201-216. doi:10.1002/sim.4372

Hennessy EA, Johnson BT, Keenan C. Best Practice Guidelines and Essential Methodological Steps to Conduct Rigorous and Systematic Meta-Reviews. *Appl Psychol Health Well Being*. 2019;11(3):353-381. doi:10.1111/aphw.12169

Ballard M, Montgomery P. Risk of bias in overviews of reviews: a scoping review of methodological guidance and four-item checklist. *Res Synth Methods*. 2017;8(1):92-108. doi:10.1002/jrsm.1229

Pieper D, Antoine SL, Neugebauer EA, Eikermann M. Up-to-dateness of reviews is often neglected in overviews: a systematic review. *J Clin Epidemiol*. 2014;67(12):1302-1308. doi:10.1016/j.jclinepi.2014.08.008

Pollock M, Fernandes RM, Newton AS, Scott SD, Hartling L. The impact of different inclusion decisions on the comprehensiveness and complexity of overviews of reviews of healthcare interventions. *Syst Rev*. 2019;8(1):18. Published 2019 Jan 11. doi:10.1186/s13643-018-0914-3

Foisy M, Becker L, Chalmers J, Boyle R, Simpson E, Williams H. Mixing with the 'unclean’: Including non-Cochrane reviews alongside Cochrane reviews in overviews of reviews. 2011 Cochrane Colloquium, Madrid.

Pollock A, Campbell P, Brunton G, Hunt H, Estcourt L. Selecting and implementing overview methods: implications from five exemplar overviews. *Syst Rev*. 2017 Jul 18;6(1):145. doi: 10.1186/s13643-017-0534-3

Liu HX, Hu DH, Yin HQ. [Umbrella review - a new method related to evidence-based medical analysis]. *Zhonghua Liu Xing Bing Xue Za Zhi*. 2020;41(2):261-266. doi:10.3760/cma.j.issn.0254-6450.2020.02.021

Cruzat B, Reveco-Guzmán K, Encina-Meneses M, Ortiz-Muñoz L, Bracchiglione J. Approaching the body of evidence: Key concepts of Overviews. Abordando el cuerpo de evidencia: conceptos fundamentales de los overviews. *Medwave*. 2023;23(5):10.5867/medwave.2023.05.2704. Published 2023 Jun 6. doi:10.5867/medwave.2023.05.2704

Aguilera-Eguía RA, Pérez-Galdavini V, Fuentes-Barría H, Roco-Videla Á. ¿Cuándo se justifica la realización de un resumen de revisiones sistemáticas (overview)? [When is a summary of systematic reviews (overview) justified?]. *Nutr Hosp*. 2023;40(3):680-681. doi:10.20960/nh.04619

Piso B, Semlitsch T, Reinsperger I, Breuer J, Kaminski-Hartenthaler A, Kien C, Thaler K, Siebenhofer A. *Z Evid Fortbild Qual Gesundhwes*. 2015;109(4-5):300-308. doi: 10.1016/j.zefq.2015.06.007

Pamporis K, Bougioukas KI, Karakasis P, Papageorgiou D, Zarifis I, Haidich AB. Overviews of reviews in the cardiovascular field underreported critical methodological and transparency characteristics: a methodological study based on the Preferred Reporting Items for Overviews of Reviews (PRIOR) statement. J Clin Epidemiol. 2023;159:139-150. doi:10.1016/j.jclinepi.2023.05.018

Kolaski K, Logan LR, Ioannidis JPA. Guidance to Best Tools and Practices for Systematic Reviews. JBJS Rev. 2023;11(6):e23.00077. Published 2023 Jun 7. doi:10.2106/JBJS.RVW.23.00077

**Not umbrella review (n=17)**

Santesso N, Glenton C, Dahm P, et al. GRADE guidelines 26: informative statements to communicate the findings of systematic reviews of interventions. *J Clin Epidemiol*. 2020;119:126-135. doi:10.1016/j.jclinepi.2019.10.014

Cuello-Garcia CA, Santesso N, Morgan RL, et al. GRADE guidance 24 optimizing the integration of randomized and non-randomized studies of interventions in evidence syntheses and health guidelines. *J Clin Epidemiol*. 2022;142:200-208. doi:10.1016/j.jclinepi.2021.11.026

Hilton Boon M, Thomson H, Shaw B, et al. Challenges in applying the GRADE approach in public health guidelines and systematic reviews: a concept article from the GRADE Public Health Group*. J Clin Epidemiol*. 2021;135:42-53. doi:10.1016/j.jclinepi.2021.01.001

Ouma LO, Wason JMS, Zheng H, Wilson N, Grayling M. Design and analysis of umbrella trials: Where do we stand? *Front Med (Lausanne)*. 2022;9:1037439. doi: 10.3389/fmed.2022.1037439.

Sales PHH, Leão JC. Why perform overviews and umbrella reviews in oral and maxillofacial surgery?. *Br J Oral Maxillofac Surg*. 2021;59(1):132-133. doi:10.1016/j.bjoms.2020.08.005

Brennan SE, Johnston RV. Research Note: Interpreting findings of a systematic review using GRADE methods. *J Physiother*. 2023;69(3):198-202. doi:10.1016/j.jphys.2023.05.012

Brozek JL, Canelo-Aybar C, Akl EA, et al. GRADE Guidelines 30: the GRADE approach to assessing the certainty of modeled evidence-An overview in the context of health decision-making. *J Clin Epidemiol*. 2021;129:138-150. doi:10.1016/j.jclinepi.2020.09.018

De Santis KK, Pieper D, Lorenz RC, Wegewitz U, Siemens W, Matthias K. User experience of applying AMSTAR 2 to appraise systematic reviews of healthcare interventions: a commentary. *BMC Med Res Methodol*. 2023;23(1):63. doi:10.1186/s12874-023-01879-8

Schünemann HJ, Brennan S, Davoli M, et al. Strong and high-quality evidence synthesis needs Cochrane: a statement of support by the GRADE Guidance Group. *J Clin Epidemiol*. 2022;152:285-287. doi:10.1016/j.jclinepi.2022.10.001

Gould DJ. Commentary on: Evaluating the Quality of Systematic Reviews and Meta-analyses About Breast Augmentation Using AMSTAR. *Aesthet Surg J Open Forum*. 2021 Jun 11;3(3):ojab023. doi: 10.1093/asjof/ojab023

Chapman JR. Editorial Perspective: Time for Another Grading System-From PRISMA to AMSTAR 2. Global Spine J. 2020;10(5):674-675. doi:10.1177/2192568220920889

Cochrane Methods: Comparing multiple interventions. Editorial considerations for reviews that compare multiple interventions. Accessed from: https://methods.cochrane.org/cmi/editorial-considerations-reviews-compare-multiple-interventions

Pollock A, van Wijck F. Cochrane overviews: how can we optimize their impact on evidence-based rehabilitation?. *Eur J Phys Rehabil Med*. 2019;55(3):395-410. doi:10.23736/S1973-9087.19.05780-0

Veroniki AA, Huedo-Medina TB, Fountoulakis KN. Appraising Between-Study Homogeneity, Small-Study Effects, Moderators, and Confounders. In: Biondi-Zoccai G, editor. Umbrella Reviews. Cham: Springer; 2016. Doi:10.1007/978-3-319-25655-9_12

Ryan RE, Kaufman CA, Hill SJ. Building blocks for meta-synthesis: data integration tables for summarising, mapping, and synthesising evidence on interventions for communicating with health consumers. *BMC Med Res Methodol*. 2009;9:16. doi:10.1186/1471-2288-9-16

Pollock A, Farmer SE, Brady MC, et al. An algorithm was developed to assign GRADE levels of evidence to comparisons within systematic reviews. *J Clin Epidemiol*. 2016;70:106-110. doi:10.1016/j.jclinepi.2015.08.013

Elliott L, Crombie IK, Irvine L, Cantrell J, Taylor J. The effectiveness of public health nursing: the problems and solutions in carrying out a review of systematic reviews. *J Adv Nurs*. 2004;45(2):117-125. doi:10.1046/j.1365-2648.2003.02873.x

**Cannot access (n=21)**

Hernández-González O, Mejías BIR, González-Fernández DF. Review of reviews: a new method to address the empirical literature. *Bibl An Investig*. 2023;19(1):195-205.

Abstracts of the 25th Cochrane Colloquium. 2018;9. doi:10.1002/14651858.CD201801

Li T, Becker L. Comparing Multiple Interventions Workshop.

Cochrane Comparing Multiple Interventions. Methods Innovation Fund - Stream 1. Accessed from: https://methods.cochrane.org/cmi/methods-innovation-fund-stream-1

Holly C, Salmond S, Saimbert M. Comprehensive Systematic Review for Advanced Practice Nursing. 2nd ed. New York: Springer Publishing Company; 2016.

Booth AM, Jones-Diette JS. Registering the Review. *Diagnostic Meta-Analysis*. Springer. 2018. p.59-75 Epub 2018 May 4.

Golder S, Wright K. Searching Evidence. In: Biondi-Zoccai G, editor. Umbrella Reviews. Cham: Springer; 2016. doi:10.1007/978-3-319-25655-9_7

Biondi-Zoccai G, La Torre G, Roever L, D’Ascenzo F. Abstracting evidence. In: Biondi-Zoccai G, editor. Umbrella Reviews. Cham: Springer; 2016. doi:10.1007/978-3-319-25655-9_8

Chen TT, Tu YK. Statistical models for overviews of reviews. In: Biondi-Zoccai G, editor. Umbrella reviews. Cham: Springer; 2016. doi:10.1007/978-3-319-25655-9_10

Baker WL, Bennetts M, Coleman CI, Cappelleri JC. Appraising evidence. In: Biondi-Zoccai G, editor. Umbrella reviews. Cham: Springer; 2016. doi:10.1007/978-3-319-25655-9_9

Papageorgiou SN, Biondi-Zoccai G. Designing the review. In: Biondi-Zoccai G, editor. Umbrella reviews. Cham: Springer; 2016. doi:10.1007/978-3-319-25655-9_5

Polo FJV, Negrín MA, Martel M. Bayesian approach to evidence synthesis. In: Biondi-Zoccai G, editor. Umbrella reviews. Cham: Springer; 2016. doi:10.1007/978-3-319-25655-9_11

Flodgren G, Shepperd S, Eccles M. Challenges facing reviewers preparing overviews of reviews. 2011 Cochrane Colloquium, Madrid.

Pantoja T, Opiyo N, Ciaponni A, Herrera C, Lewin S, Oxman A, Paulsen E, Rada G, Wiysonge C. Strategies for improving health systems in low-income countries: lessons learnt from four overviews of systematic reviews of health systems interventions. 2015 Cochrane Colloquium, Vienna.

Kramer S, Langendam M, Elbers R, Scholten R, Hooft L. Preparing an overview of reviews: lessons learned. 2009 Cochrane Colloquium, Singapore.

Rojas M, Lozano J, Solà I, Bonfill X. Incorporating the GRADE approach in overviews of systematic reviews: an example from an overview in neonatal respiratory care. 2011 Cochrane Colloquium, Madrid.

Shepherd E, Middleton P, Crowther C. Challenges of overviews of reviews and how to overcome them, informed by a public health overview. 2016 Cochrane Colloquium, Seoul.

Bofill Rodriguez M, Jordan V, Lethaby A, Wise M, Farquhar C. How hard is to do an overview of Cochrane Reviews? Could CORE outcomes help? 2019 Cochrane Colloquium, Santiago.

Lewin S, Oxman A, Ciapponi A, Herrera C, Opiyo N, Pantoja T, Paulsen E, Rada G, Wiysonge C. Novel approaches to conducting overviews of reviews: Lessons from four overviews of health systems interventions. 2017 Cochrane Colloquium, Cape Town.

Sun C, Zhang H, Liu X, Yan S. *Chin. J. Evid.-Based Med.* 2022;22(5):609-614. doi: 10.16420/j.issn.0513-353x.2021-0059

Giannakou K, Galanis P. *Arch. Hell. Med.* 2020;37(1):129-134.

**Not guidance/author’s experience (n=15)**

Bonczar M, Ostrowski P, D'Antoni AV, Tubbs RS, Iwanaga J, Ghosh SK, Klejbor I, Kuniewicz M, Walocha J, Morys J, Koziej M. How to write an umbrella review? A step-by-step tutorial with tips and tricks. *Folia Morphol (Warsz)*. 2023;82(1):1-6. doi:10.5603/FM.a2022.0104

Wang R, Papageorghiou AT. Umbrella review-a useful tool when it's raining systematic reviews. *BJOG*. 2021;128(13):2150. doi:10.1111/1471-0528.16827

Choi GJ, Kang H. The umbrella review: a useful strategy in the rain of evidence. *Korean J Pain*. 2022;35(2):127-128. doi: 10.3344/kjp.2022.35.2.127

Yang N, Liu H, Zhang K, Chen Y, Estill J. Viewpoints on the PRIOR statement-a reporting guideline for overviews of reviews. *Ann Transl Med*. 2023;11(5):230. doi:10.21037/atm-22-5724

Lorenz RC, Matthias K, Pieper D, Wegewitz U, Morche J, Nocon M, Rissling O, Schirm J, Freitag S, Jacobs A. AMSTAR 2 overall confidence rating: lacking discriminating capacity or requirement of high methodological quality?. *J Clin Epidemiol*. 2020;119:142-144. doi:10.1016/j.jclinepi.2019.10.006

Arian M, Valindejadi A, Soleimani M. Calculate the actual overlap in an overview. Nursing Practice Today. 2021;8(2). doi:10.18502/npt.v8i2.5119

Mistiaen P, Leroy R, Van de Voorde C, Stordeur S, Van den Heede K. HSR Process Notes: Literature review and international comparison. Method Brussels: Belgian Health Care Knowledge Centre (KCE). 2016.

Becker L, Thomson D, Caldwell D. Addressing Multiple Treatments I: Cochrane Overviews. Accessed from: https://methods.cochrane.org/sites/methods.cochrane.org.cmi/files/public/uploads/Becker%20-%20Intro%20to%20Overviews%20Keystone.pdf

Pérez-Bracchiglione J, Niño de Guzmán E, Roqué Figuls M, Urrútia G. Graphical representation of overlap degree of primary studies in systematic reviews included in overviews. 2019 Cochrane Colloquium, Santiago.

Hartling L, Pollock M, Fernandes R, Brennan S. Integrating evidence across reviews: an introduction to overviews of reviews. 2016 Cochrane Colloquium, Seoul.

Cooper H, Koenka AC. The overview of reviews: unique challenges and opportunities when research syntheses are the principal elements of new integrative scholarship. *Am Psychol*. 2012;67(6):446-462. doi:10.1037/a0027119

Conn VS, Coon Sells TG. WJNR welcomes umbrella reviews. *West J Nurs Res*. 2014;36(2):147-151. doi:10.1177/0193945913506968

Pieper D, Antoine SL, Mathes T, Neugebauer EA, Eikermann M. Systematic review finds overlapping reviews were not mentioned in every other overview. *J Clin Epidemiol*. 2014;67(4):368-375. doi:10.1016/j.jclinepi.2013.11.007

Thomson D, Foisy M, Oleszczuk M, Wingert A, Chisholm A, Hartling L. Overview of reviews in child health: evidence synthesis and the knowledge base for a specific population. *Evid Based Child Health*. 2013;8(1):3-10. doi:10.1002/ebch.1897

Misfeldt R, Hepp S. Research design of overview of reviews: incentives for health care providers. In: Sage research methods cases part 2. London: SAGE Publications, Ltd.; 2017. doi:10.4135/9781526409706

**Appendix S2.** Documents included in the scoping review.

**Documents included in the scoping review (n=8).**

Bougioukas KI, Pamporis K, Vounzoulaki E, Karagiannis T, Haidich AB. Types and associated methodologies of overviews of reviews in health care: a methodological study with published examples. J Clin Epidemiol. 2023 Jan;153:13-25.

Aromataris E, Fernandez R, Godfrey CM, Holly C, Khalil H, Tungpunkom P. Summarizing systematic reviews: methodological development, conduct and reporting of an umbrella review approach. Int J Evid Based Healthc. 2015 Sep;13(3):132-40. doi: 10.1097/XEB.0000000000000055.

Baker PR, Costello JT, Dobbins M, Waters EB. The benefits and challenges of conducting an overview of systematic reviews in public health: a focus on physical activity. J Public Health (Oxf). 2014 Sep;36(3):517-21. doi: 10.1093/pubmed/fdu050. Epub 2014 Aug 1.

Belbasis L, Bellou V, Ioannidis JPA. Conducting umbrella reviews. BMJ Med. 2022 Nov 22;1(1):e000071. doi: 10.1136/bmjmed-2021-000071.

Fusar-Poli P, Radua J. Ten simple rules for conducting umbrella reviews. Evid Based Ment Health. 2018 Aug;21(3):95-100. doi: 10.1136/ebmental-2018-300014.

Gianfredi V, Nucci D, Amerio A, Signorelli C, Odone A, Dinu M. What Can We Expect from an Umbrella Review? Adv Nutr. 2022 Mar;13(2):684-685. doi: 10.1093/advances/nmab150.

Arango C, Dragioti E, Solmi M, Cortese S, Domschke K, Murray RM, Jones PB, Uher R, Carvalho AF, Reichenberg A, Shin JI, Andreassen OA, Correll CU, Fusar-Poli P. Risk and protective factors for mental disorders beyond genetics: an evidence-based atlas. World Psychiatry. 2021 Oct;20(3):417-436. doi: 10.1002/wps.20894.

Janiaud P, Agarwal A, Tzoulaki I, Theodoratou E, Tsilidis KK, Evangelou E, Ioannidis JPA. Validity of observational evidence on putative risk and protective factors: appraisal of 3744 meta-analyses on 57 topics. BMC Med. 2021 Jul 6;19(1):157. doi: 10.1186/s12916-021-02020-6.
